# Supplementary material for: Cognitive fatigability and neuronal correlates in chronic pain – A cross-sectional fMRI study
Source: PLoS One. 2025 Nov 17;20(11):e0332799. doi: 10.1371/journal.pone.0332799 (PMC12622801; doi:10.1371/journal.pone.0332799)
Supplement: S1 File — (DOCX) [file pone.0332799.s001.docx]

Link for access to fMRI data used in” Cognitive fatigability and neuronal correlates in chronic pain – a cross-sectional fMRI study”

<https://zenodo.org/records/17341991?token=eyJhbGciOiJIUzUxMiJ9.eyJpZCI6IjBmMmYzOTZkLTVmZTctNDg5Zi1hZmY5LWNmOWYzZGYwNjVlZiIsImRhdGEiOnt9LCJyYW5kb20iOiIwZjkwNzg1Yzc4NzEzNjcxMTdkYTRjZTc4M2E5Yzc0MiJ9.-2vW5wBWGNaG_vHsv_VLEL1jswRjoOUVKcTvvEYrD_G2RCcDx5nhp0ez6mFED8kxJfLatX8tuj3U5RPSv9S1lw>
